# Energetics and mechanics of kicking with lifeguarding fins

**DOI:** 10.3389/fspor.2026.1841097

**Published:** 2026-06-09

**Authors:** Harrison C. Thomas, Isabella O. Martinez, Sean C. Newcomer, Jeff A. Nessler, George H. Crocker

**Affiliations:** 1 Department of Kinesiology, California State University, San Marcos, CA, United States; 2 School of Kinesiology, California State University, Los Angeles, CA, United States

**Keywords:** aquatics, calorimetry, fins, lifeguarding, swimming

## Abstract

Open-water lifeguarding is a physically demanding profession, especially at crowded beaches and in dynamic ocean environments. Lifeguards often use fins when performing rescues as they can improve performance and decrease fatigue. The purpose of this study is to compare the energetics and mechanics of kicking with three different pairs of lifeguarding fins (*DaFiN Pro Classic, Viper V7, Duck Feet*) to a pair of swim fins (*Arena Powerfin Pro*). Thirty-one lifeguards (22 men, 9 women; 24 ± 6 years old) completed a maximal graded kicking test in a swim flume wearing the *Arena Powerfin Pro*, followed by four randomized 5-min submaximal trials at ∼80% of their peak kicking speed (0.93 ± 0.17 m/s). Cost of kicking was significantly lower in *DaFiN Pro Classic* (684 ± 156 J/m) and *Duck Feet* (663 ± 156 J/m) compared to the swim fins (780 ± 145 J/m), but not with the *Viper V7* fins (733 ± 152 J/m). Kicking intensity was reduced with all three lifeguarding fins relative to the swim fins and the *Duck Feet* fins had a reduced kicking intensity relative to the *Viper V7* fins. All lifeguard fins elicited a lower kick cadence than the swim fins and there were no differences in cadence among the lifeguarding fins. These findings suggest that lifeguard-specific fins have metabolic and biomechanical advantages relative to swim fins. Lifeguard agencies should consider kicking mechanics and energetics, alongside subjective measures, when selecting fins for their lifeguards.

## Introduction

The United States Lifesaving Association reported 152 drownings across 122 agencies in 2024 (1). However, the majority of these drownings occurred in unpatrolled areas. In patrolled beach areas, lifeguards reduce drowning risks through prevention efforts, performing rescues, and administering pre-hospital emergency care (2). The most basic rescue equipment a lifeguard possesses are a rescue tube or can and a pair of fins. In a controlled setting, fins reduce time during a 25-m kick while carrying a mannikin (3). During simulated rescues, the use of fins reduces the time to reach the victim and the time to tow them into shore, despite similar physiological exertion by the lifeguard (4–6).

Not only is speed important when making rescues, the benefit of wearing fins also lies in the reduction of the energetic costs associated with performing rescues. Therefore, research into swimming or kicking economy (*i.e.,* energy expended relative to distance traveled) with fins is necessary. Lifeguards typically approach the victim swimming head-up to maintain visual contact, a position that increases frontal drag and alters stroke mechanics compared to head-down swimming, resulting in a higher energetic cost of locomotion (7). Kinematic changes have also been observed in water polo players when swimming with their head above water (8). These metabolic and stroke differences further highlight the importance of fin selection in reducing overall metabolic cost during rescues. Swimming with fins reduces the energetic cost of swimming by ∼40% due to a slight decrease in kick amplitude and a large decrease in kick frequency (9). In addition, dolphin kicking (*i.e.,* kicking with both legs together as seen in the butterfly swimming stroke) with fins decreased energetic cost by ∼60% compared to barefoot kicking (10).

Fin design may also play a role in lifeguard performance. Rescue times were reduced and arm fatigue was lower when lifeguards used long fins compared to short fins during a simulated rescue (6). A recent study found differences among SCUBA diving fins in measures of physiological exertion at slow speeds typical of SCUBA diving and snorkeling (11). However, there was no difference in towing velocity between flexible and fiber fins during a 25-m manikin carry (3). Furthermore, research on kicking economy and mechanics with fins commonly used by lifeguards is lacking as the research of swimming or kicking economy is limited to fins used in SCUBA diving or swim training.

Fins used by lifeguards tend to be more rigid than swimming or SCUBA diving and snorkeling fins. As for surface area, lifeguarding fins are similar to swim fins, but tend to be much smaller than SCUBA diving and snorkeling fins. Lifeguard fins also have open heels that allow them to be carried easily in one hand and can be easily put on when performing a rescue, a design feature similar to some, but not all, swim fins. To our knowledge, no study has investigated the energetic cost of kicking or swimming while wearing commonly-used lifeguarding fins. Therefore, the purpose of this study was to compare the metabolic cost of kicking across three pairs of commonly used lifeguard fins compared to one pair of swim fins. We hypothesized that kicking with the lifeguard fins would reduce metabolic cost when compared to swim fins due to their larger surface areas.

## Methods

### Participants

Thirty-one active or retired open-water lifeguards (22 men and 9 women) participated in this study (Table 1). Participants ranged in age from 18 to 39 years old (24 ± 6 years old). Inclusion criteria for this study was at least one season of open-water lifeguarding experience and at least five years of swimming experience. Participants with two or more risk factors for cardiovascular disease as determined by the AHA/ACSM Health/Fitness Facility Pre-participation Screening Questionnaire were excluded from this study. Participants abstained from any intense exercise for 24 h with the exception of their occupational duties. They also abstained from caffeine for three hours and from large meals for two hours before the study. Written informed consent was obtained prior to any participation in the study. This study was approved by the California State University, San Marcos Institutional Review Board (protocol #1971178-1).

**Table 1:** Demographics, open-water lifeguarding (OWLG) experience, and swimming experience for the 31 participants (22 men & 9 women) in this study. Data are reported as means ± standard deviations with ranges in parentheses.

Height (cm)	Mass (kg)	Age (yr)	OWLG Experience (yr)	Swimming Experience (yr)
177 ± 9 (157–193)	73.1 ± 13.1 (44.5–95.5)	24 ± 6 (18–39)	3 ± 2 (1–8)	17 ± 7 (8–35)

### Fins

**Figure 1:**
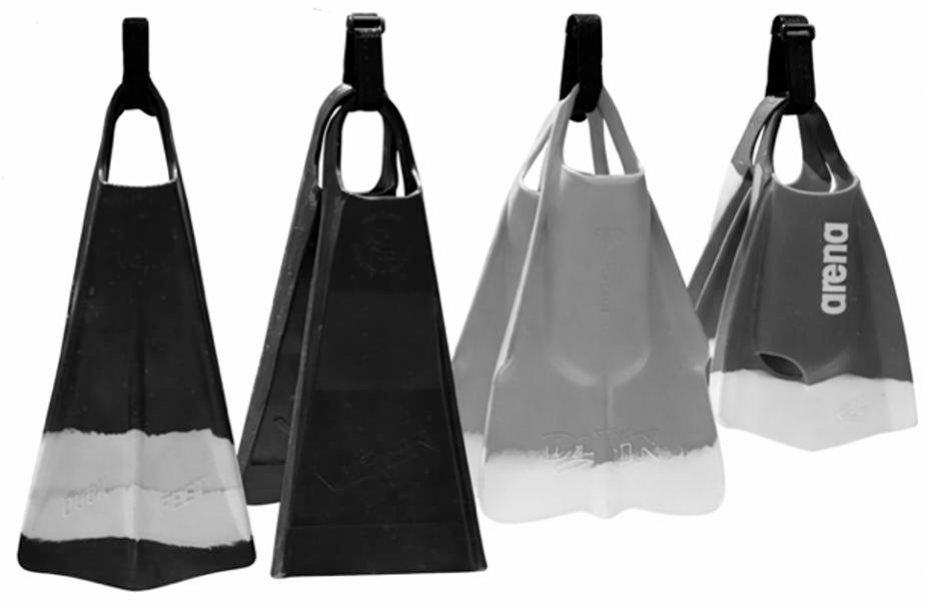
Images of the fins used in the study. From left to right: *Duck Feet, Viper V7, DaFiN Pro Classic, & Arena Powerfin Pro*.

Three pairs of lifeguarding fins and one pair of swimming fins were tested in this study (Figure 1). The three pairs of lifeguarding fins were the *DaFiN Pro Classic* (DaFiN Hawaii, Honolulu, HI, USA), *Viper V7* (Viper Surfing Fins, Newport Beach, CA, USA), and *Duck Feet* (Voit Corporation, San Antonio, TX, USA). These fins were included in this study since they are marketed towards and commonly used by lifeguards. The swimming fins were the *Arena Powerfin Pro* (Arena, Tolentino, MC, Italy) and these fins were used as the control condition. No barefoot kicking was included because barefoot kicking is much (∼60%) slower than kicking with fins (10). Participants reported their shoe size to determine their fin size according to each manufacturer's specifications.

**Figure 2:**
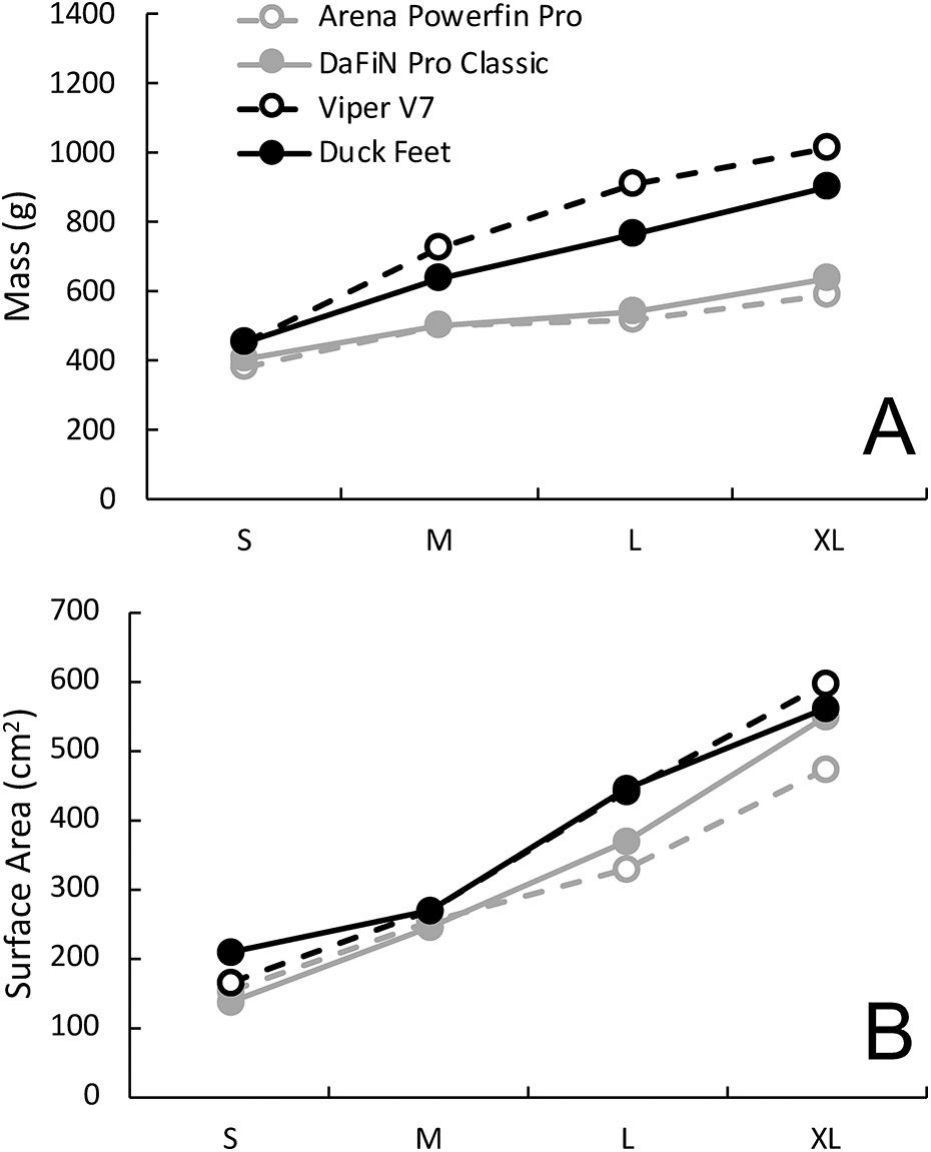
Mass (panel **A**) and anterior surface area (panel **B**) of the four different sizes (S, M, L, XL) for the four different fins. Mass and surface area were determined from a single fin.

Two-dimensional surface area of each fin was measured using *ImageJ* software (National Institutes of Health, Bethesda, MD, USA) from photographs taken using a Canon EOS Rebel T7 camera (Canon, Ōta, NRT, Japan), with a 12 in (30.5 cm) ruler for scale. Single fin weight was measured by placing an individual fin in the center of a benchtop scale (Mettler-Toledo International, Greifensee, ZRH, Switzerland). The *Viper V7* and *Duck Feet* fins were the heaviest while all three lifeguarding fins had larger surface areas related to the swim fins (Figure 2).

### Procedures

Participants arrived at a shaded, outdoor research facility that housed a swim flume (Endless Pools Elite, Aston, PA, USA). The swim flume is 4.9 by 2.7 m, with a water depth of 1.1 m, and has a speed range of 0.2–2.3 m/s. The water temperature was maintained between 24 and 25 °C (75–77°F). To warm up and familiarize themselves with the flume, participants kicked in the swim flume while wearing the *Arena Powerfin Pro* fins at a speed of 0.5 m/s for up to ten minutes. Participants used a kickboard (EVA kickboard, Nike, Beaverton, OR, USA) and kicked with their head above water for warm up and all experimental trials. Following warm up and familiarization, participants were fitted with an oronasal mask (Hans Rudolph, Inc., Shawnee, KS, USA) using the manufacturer's mask sizing calipers. The mask was affixed to a two-way non-rebreathing valve (Hans Rudolph, Inc., Shawnee, KS, USA) connected to inflow and outflow hoses (35 mm ID; 4.57 m long) situated above the surface of the water. The expiratory hose was connected to a metabolic cart with a 4-L mixing chamber (ParvoMedics TrueOne 2400, Sandy, UT, USA) to collect realtime, metabolic measurements.

To assess their peak rate of O_2_ consumption (VO_2_peak), participants performed a graded exercise test in the swim flume while wearing the *Arena Powerfin Pro* fins. The graded exercise test began at 0.5 m/s and increased by 0.1 m/s every minute until volitional fatigue, which was defined as the participant being unable to maintain a kickboard position within 30 cm from the front of the flume as marked by a tethered tennis ball. The participants then rested for 20 min after the completion of the graded exercise test.

During the 20-min rest period, participants were instrumented with two Inertial Measurement Units (IMUs, Vicon Blue Trident, Oxford, UK) placed 10.2 cm above the medial malleolus and reinforced using a transparent dressing (3M™ Tegaderm™, St. Paul, MN, USA) and then further held in place with neoprene wetsuit sleeves. Participants kicked with each pair of fins for 5 min with 10 min rest between trials. The order of these trials was randomized for each participant. The pace for all trials was 80% of their maximum speed achieved during their graded exercise test. This intensity was selected to ensure submaximal, steady-state conditions for assessment of energy expenditure via indirect calorimetry. During these trials, metabolic measures were taken as described above for the graded exercise test.

Rates of O_2_ consumption (VO_2_) and CO_2_ production (VCO_2_) were recorded continuously during each trial. Metabolic data for the last two minutes of each 5-min trial were used for analyses. Rate of energy expenditure (EE, in J/s) was calculated from VO_2_ and VCO_2_ (in L/min) using the Weir equation (12):

$$\mathrm{EE}\;=\;[\left(3.9\;\times {\;\mathrm{VO}}_{2})\;+\;(1.1\;\times {\;\mathrm{VCO}}_{2}\right)]\;*\;4184\;/\;60$$

Cost of kicking (in J/m) was then calculated by dividing EE by kicking speed. Kicking speed was determined from a flowmeter (JDC Electronics, Yverdon-les-Bains, VD, Switzerland) placed 5 cm from the flume's grate.

The IMUs sampled at 1125 Hz and data from the full 5-min trial were used for analyses. Kick duration was determined as the average time between successive peaks using filtered accelerometer data (fourth order Butterworth, with 2 Hz cutoff). Kick cadence was calculated as the inverse of kick duration. Acceleration was determined from the raw, unfiltered data by first calculating the magnitude of acceleration using acceleration vectors in the X, Y, and Z directions. Kick intensity was then calculated as the average of acceleration peaks across all kicks. Any artifact in the acceleration data that was more than 2.5 standard deviations above the average acceleration peak was removed prior to calculating trial averages.

Participants were given a paper form listing all four pairs of fins by name. For comfort, each participant independently assigned a rank from 1 (most comfortable) to 4 (least comfortable) to each pair. They then completed a separate ranking for ease of kicking, again assigning ranks from 1 (easiest) to 4 (hardest). Rankings were completed individually and without discussion among participants. No additional definitions of comfort or ease of kicking were provided beyond the terms themselves, allowing participants to interpret these constructs based on their own experience.

### Statistical analysis

Data are reported as means and standard deviations. Repeated-measures analysis of variance was used to determine if differences existed among pairs of fins for any measured endpoint. Sphericity was assessed using Mauchly's test for sphericity. The Greenhouse-Geisser correction was applied when the sphericity assumption was violated. Pairwise comparisons between pairs of fins were made using Bonferroni's correction for multiple comparisons. Data was analyzed using IBM SPSS Statistics 29 (IBM, Armonk, NY, USA) with α of 0.05.

## Results

The graded exercise test while kicking with the swim fins (*Arena Powerfin Pro*) elicited a VO_2_peak of 40.9 ± 7.5 mL kg^−1^ min^−1^, which corresponded to a current speed of 1.14 ± 0.19 m s^−1^ (equivalent to 88 ± 11 s per 100 m). The RER at VO_2_peak was 1.13 ± 0.11. Mean current speed during steady-state exercise trials was 0.93 ± 0.17 m s^−1^, which is equivalent to 108 ± 10 s per 100 m and 81.5 ± 3.1% of the speed eliciting VO_2_peak. This pace elicited a VO_2_ with the swim fins of 28.6 ± 5.3 mL kg^−1^ min^−1^ that is equivalent to 71.1 ± 12.5% VO_2_peak when kicking with the swim fins.

**Figure 3:**
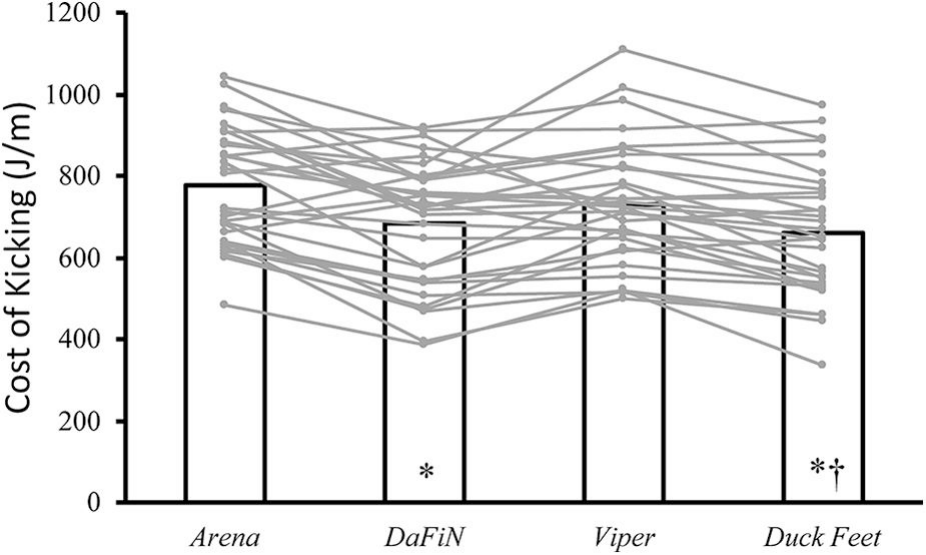
Cost of kicking across the fins used in this study. Bars denote the average of all participants and connected gray lines indicate individual participants. * denote significantly different from *Arena Powerfin Pro*; † denote significantly different from *Viper V7* fins (*p* < 0.05).

When kicking with the swim fins, VO_2_ and VCO_2_ were 2.05 ± 0.50 L min^−1^ and 2.06 ± 0.64 L min^−1^, respectively (Table 2). All three pairs of lifeguarding fins reduced VO_2_ and VCO_2_ at the same submaximal speed. Energy expenditure averaged 717 ± 183 J s^−1^ for the swim fins and decreased significantly with all three pairs of lifeguarding fins. Cost of kicking was 780 ± 145 J m^−1^ and was significantly reduced in two of the three lifeguarding fins (except *Viper V7* fins, *p* = 0.098; Figure 3). In addition, the *Duck Feet* fins had a lower cost of kicking compared to the *Viper V7* fins. While kicking with the swim fins, RER was 0.99 ± 0.08, which was also significantly higher than the two of the three lifeguarding fins (except *Viper V7* fins, *p* = 0.064).

**Table 2:** Rate of O_2_ consumption (VO_2_), rate of CO_2_ production (VCO_2_), respiratory exchange ratio (RER), rate of energy expenditure (EE), cost of kicking (CK), kick cadence and kick intensity with lifeguard fins for all 31 participants.

Measurement	*Arena Powerfin Pro*	*DaFiN Pro Classic*	*Viper V7*	*Duck Feet*
VO_2_ (L/min)	2.05 ± 0.50	1.82 ± 0.48^a^	1.93 ± 0.42^a^	1.77 ± 0.46^a^^,^^b^
VCO_2_ (L/min)	2.06 ± 0.64	1.71 ± 0.59^a^	1.85 ± 0.44^a^	1.62 ± 0.51^a^^,^^b^
RER	0.99 ± 0.08	0.93 ± 0.08^a^	0.95 ± 0.07	0.91 ± 0.07^a^^,^^b^
EE (J/s)	717 ± 183	627 ± 176^a^	667 ± 148^a^	605 ± 164^a^^,^^b^
CK (J/m)	780 ± 145	684 ± 156^a^	733 ± 152	663 ± 156^a^^,^^b^
Kick Cadence (min^−1^)	71.1 ± 14.1	64.0 ± 13.9^a^	65.7 ± 18.7^a^	61.9 ± 14.5^a^
Kick Intensity (m/s^2^)	21.5 ± 2.7	19.6 ± 2.6^a^	20.3 ± 2.8^a^	19.0 ± 2.6^a^^,^^b^

a denote significantly different (*p* < 0.05) from the *Arena Powerfin Pro* fins.

b denote significantly different (*p* < 0.05) from the *Viper V7* fins.

**Figure 4:**
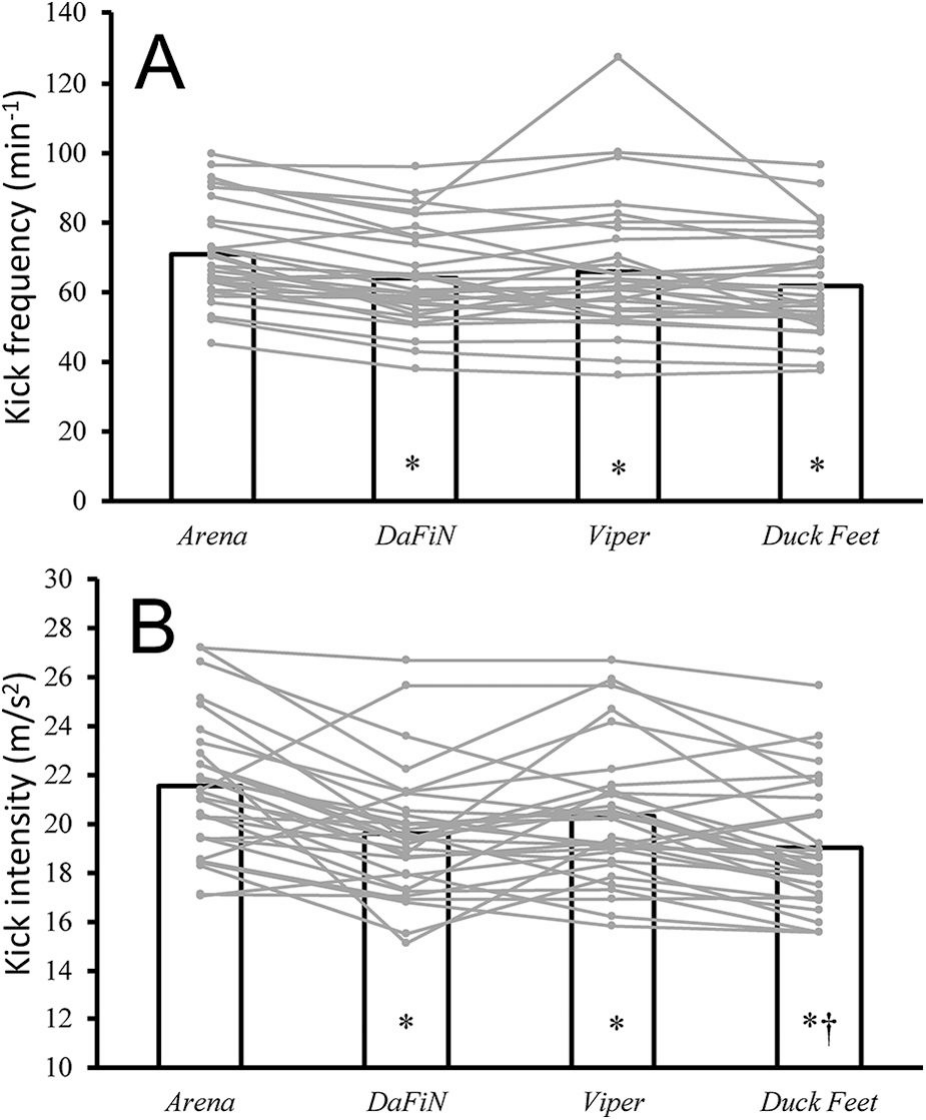
Kicking frequency (panel **A**) and kicking intensity (panel **B**) across the fins used in this study. Bars denote the average of all participants and connected gray lines indicate individual participants. * denote significantly different from *Arena Powerfin Pro*; † denote significantly different from *Viper V7* fins (*p* < 0.05).

Kick cadence with the swim fins was 71.1 ± 14.1 min^−1^ and decreased significantly when wearing each pair of lifeguarding fins (Figure 4). Similarly, kick intensity was 21.5 ± 2.7 m s^−2^ and decreased significantly when wearing each pair of lifeguarding fins. In addition, the *Duck Feet* fins had a lower kicking intensity compared to the *Viper V7* fins.

Perceptually, participants ranked *DaFiN Pro Classic* as the most comfortable, followed by *Duck Feet*, then *Arena Powerfin Pro*, and lastly the *Viper V7* (Table 3). For ease of use, *Duck Feet* were ranked as the easiest, followed by *DaFiN Pro Classic*, *Arena Powerfin Pro*, and lastly the *Viper V7* (Table 4).

**Table 3:** Participants ranking fins in order of comfort. Scores reflect the mean of all participants’ rankings.

Fin	1st	2nd	3rd	4th	Score
*DaFiN Pro Classic*	17	9	4	1	1.65
*Duck Feet*	8	11	10	2	2.19
*Arena Powerfin Pro*	6	11	11	3	2.35
*Viper V7*	0	0	6	25	3.81

**Table 4:** Participants ranking fins in order of ease of kicking. Scores reflect the mean of all participants’ rankings.

Fin	1st	2nd	3rd	4th	Score
*Duck Feet*	16	11	4	0	1.61
*DaFiN Pro Classic*	13	10	7	1	1.87
*Arena Powerfin Pro*	2	7	12	10	2.97
*Viper V7*	0	3	8	20	3.55

## Discussion

The major finding of this study was that different lifeguarding fins affect the energetic cost of kicking, kicking cadence, and kicking intensity during submaximal, steady-state kicking. For the energetic cost of kicking, the *DaFiN Pro Classic* and *Duck Feet* fins both elicited a significantly lower cost of kicking compared to the *Arena Powerfin Pro* swim fins, while the *Viper V7* fins did not differ significantly from the swim fins. Therefore, our hypothesis that lifeguard fins would reduce the metabolic cost of kicking relative to swim fins was partially supported. All lifeguarding fins had lower kicking intensity and kick cadence relative to the swim fins. To our knowledge, this is the first study to quantify the energetics and mechanics of kicking with fins commonly used by lifeguards.

The reduction in cost of kicking with the *DaFiN Pro Classic* and *Duck Feet* fins aligns with previous research on snorkeling/SCUBA and swimming fins, which suggests that larger fin surface area reduces the energetic cost of kicking (9–11). However, the differences in surface area were more pronounced across sizes within each fin model than across fin models themselves. Fin mass differed more substantially across models. The *Duck Feet* and *Viper V7 fins* were heavier relative to the *DaFiN Pro Classic* and the *Arena Powerfin Pro* swim fins. However, the *Duck Feet* and *DaFiN Pro Classic* were the most economical when kicking, suggesting that mass on land does not determine energetic cost.

The interaction between surface area, density, stiffness, and blade geometry likely determines each fin pair's kicking economy. A recent study showed that stiffer fins increased energetic costs (11), which could be explained by greater muscle recruitment of larger, more proximal leg muscle groups (9, 11). In addition, stiffer fins tend to produce deeper kicks with higher drag, while more flexible fins are associated with higher kick frequencies and lower efficiency at those frequencies (13, 14). Therefore, the differences in the energy costs in the present study could be due to differences in fin stiffness causing different lower body muscle recruitment patterns (11), although these measurements were not taken. Lower kick frequency is also associated with lower energetic costs (13), which suggests that the slower kick cadence with lifeguarding fins may contribute to the lower energetic cost.

Kick cadence and kicking intensity were lower with all three lifeguarding fins compared to the swim fins. Because kicking speed was held constant across all three trials, the reduced kicking frequency corresponds with a greater distance per kick with the lifeguarding fins relative to the swim fins. The slower kicking cadence with the lifeguarding fins explains the reduced acceleration as the velocity of each limb is changing at a slower rate. This coupling between cadence and acceleration is mechanically expected. A slower kick cycle produces lower peak angular velocities of the shank, directly reducing the acceleration magnitude captured by the IMUs. However, the IMUs were placed only on the shank and captured overall limb acceleration. Multiple IMUs would have been needed to isolate contributions from individual joints such as the ankle, knee or hip.

Much of the research on kick cadence with fins is limited to those common in SCUBA diving. These fins tend to be long and flexible to reduce energetic costs by limiting the recruitment of the larger, more proximal leg muscles (11). Lower energetic costs enable SCUBA divers to consume their air supply more slowly and extend their dive duration. Swimmers kick faster and more forcefully than SCUBA divers as they are more concerned with forward propulsion and reducing drag, rather than reduced oxygen consumption and minute ventilation (9). Therefore, they tend to kick primarily with their hip flexors and extensors as well as their ankle plantar flexors and dorsiflexors (11). Swim fins tend to be much smaller than SCUBA diving fins and are often very flexible, though some are small and rigid. There has also been research on the monofin, a large, rigid single fin where swimmers are limited to the dolphin kick common in the butterfly stroke because both feet attach to the single fin (10, 15). Lifeguarding fins tend to be stiffer than swim fins. These fins are also marketed towards bodysurfers and bodyboarders whose kick pattern is short bursts of explosive kicking to catch waves mixed with long periods of slow kicking and rest. These fins are likely popular with lifeguards as they provide the greatest propulsion which is needed with the added resistance when swimming with their head out of the water and when towing the victim to shore.

Lifeguards may also consider subjective measurements, such as ease of kicking and comfort, when selecting a pair of fins. Participants ranked *Duck Feet* as the easiest to kick with, followed by *DaFiN Pro Classic*, *Arena Powerfin Pro*, and lastly *Viper V7*. One must consider the task when interpreting these rankings. Kicking at 80% of the speed eliciting VO_2_peak with fins for five minutes is different from the dynamic, high-intensity demands of an open-water rescue. The relative alignment of ease of kicking and kicking economy for the fins in this study suggests that lifeguard subjective preference may predict energetic cost in field settings where laboratory measurements are not feasible. Comfort rankings favored the *DaFiN Pro Classic*, followed by *Duck Feet*, *Arena Powerfin Pro*, and *Viper V7*. These rankings likely reflect the influence of factors, such as fin surface area, stiffness, density, design and foot-pocket fit.

The use of the swim flume provided many advantages including the ability to control the pace at which each lifeguard kicked. This enabled the intensity to be set at a similar relative intensity (∼80% VO_2_peak speed) despite differences in VO_2_peak and speed at VO_2_peak among participants. In addition, the use of the swim flume kept the participant stationary which enabled metabolic measurements. Despite these benefits, there are also limitations from using a swim flume, primarily related to ecological validity. Lifeguard rescues occur at near-maximal intensities, particularly during the initial sprint to reach the victim (4–6). This may lead to different muscle recruitment patterns and the onset of fatigue, leading to changes in speed and kicking mechanics. In addition, participants kicked with a kickboard at a constant, controlled intensity, but did not replicate the dynamic demands of open-water rescues. Future studies could examine these fins during simulated open-water rescues.

Fin stiffness and other mechanical properties were not assessed, and both may influence kicking mechanics and cost of kicking (10, 11). Additionally, because this study evaluated only commercially available lifeguard fins rather than purpose-built experimental prototypes, individual design characteristics such as stiffness, blade compliance, and material composition could not be isolated or manipulated independently. Future studies should isolate these variables systematically, using purpose-designed experimental fins that allow individual characteristics to be varied independently. In addition, future studies could incorporate electromyography or joint-level kinematics to help clarify the specific biomechanical and neuromuscular mechanisms underlying the differences in metabolic costs when swimming or kicking with different fins.

Given that aquatic rescues are physiologically demanding (4, 5), selecting fins that minimize the cost of kicking could reduce fatigue and improve performance in open-water lifeguards. This may be especially important for lifeguards at busier beaches where rescues are performed more frequently and at locations where lifeguards must swim longer distances to reach the victims. More broadly, understanding the relationship between fin design and kicking economy could inform the development of fins specifically tailored to the demands of the open-water lifeguards. Future research should isolate individual fin characteristics (*i.e.,* surface area, stiffness, mass, design) to fully elucidate the relationship between fin design and rescuer performance, with the ultimate goal of optimizing lifeguard equipment that can contribute to saving lives.

## Data Availability

The raw data supporting the conclusions of this article will be made available by the authors, without undue reservation.
